# Comparative Evaluation of *β*-Cyclodextrin Inclusion Complexes with Eugenol, Eucalyptol, and Clove Essential Oil: Characterisation and Antimicrobial Activity Assessment for Pharmaceutical Applications

**DOI:** 10.3390/pharmaceutics17070852

**Published:** 2025-06-29

**Authors:** Alina Ionela Stancu, Magdalena Mititelu, Anton Ficai, Lia-Mara Ditu, Mihaela Buleandră, Irinel Adriana Badea, Elena Pincu, Marius Constantin Stoian, Oana Brîncoveanu, Adina Boldeiu, Eliza Oprea

**Affiliations:** 1 Department Science and Engineering of Oxide Materials and Nanomaterials, Faculty of Chemical Engineering and Biotechnology, National University of Science and Technology Politehnica Bucharest, 1-7 Polizu Street, 011061 Bucharest, Romania; alina.stancu1995@gmail.com; 2 Department of Clinical Laboratory and Food Safety, Faculty of Pharmacy, University of Medicine and Pharmacy Carol Davila, 6 Traian Vuia Street, 020956 Bucharest, Romania; 3 Academy of Romanian Scientists, Ilfov Street 1-3, 050044 Bucharest, Romania; 4 Department of Botany and Microbiology, Faculty of Biology, University of Bucharest, Portocalilor 1-3, 77206 Bucharest, Romania; lia-mara.ditu@bio.unibuc.ro (L.-M.D.); eliza.oprea@g.unibuc.ro (E.O.); 5 Research Institute of the University of Bucharest—ICUB, University of Bucharest, Splaiul Independentei 91-95, 050657 Bucharest, Romania; 6 Department of Analytical Chemistry and Physical Chemistry, Faculty of Chemistry, University of Bucharest, 90-92 Panduri Street, 050663 Bucharest, Romania; mihaela.buleandra@g.unibuc.ro (M.B.); irinel.badea@chimie.unibuc.ro (I.A.B.); elena.pincu@chimie.unibuc.ro (E.P.); 7 National Institute for Research and Development in Microtechnologies, 126A Erou Iancu Nicolae Street, Voluntari City, Ilfov County, 077190 Bucharest, Romania; marius.stoian@imt.ro (M.C.S.); oana.brincoveanu@imt.ro (O.B.); adina.boldeiu@imt.ro (A.B.)

**Keywords:** *β*-cyclodextrin, *Eugenia caryophyllata* essential oil, eugenol, cyclodextrin inclusion complexes, antimicrobial activity

## Abstract

Clove essential oil (*Eugenia caryophyllata* essential oil, ECEO) is known for its high eugenol content and notable antimicrobial properties. However, the volatility and instability of its active compounds hinder broader pharmaceutical applications. **Methods:** This study characterised the chemical composition of ECEO and comparatively evaluated four *β*-cyclodextrin (*β*-CD) encapsulation methods: kneading, co-precipitation, lyophilisation, and co-precipitation–lyophilisation for eugenol, eucalyptol, and ECEO. Encapsulation efficiency, physicochemical properties, and antimicrobial potential were assessed. Analytical techniques included Gas Chromatography–Mass Spectrometry (GC-MS), Headspace GC-MS (HS-GC-MS), Differential Scanning Calorimetry (DSC), Job’s method, and Dynamic Light Scattering (DLS). **Results:** GC-MS identified eugenol (90.67%), eugenyl acetate (4.77%), and (E)–*β*-caryophyllene (3.98%) as major components of ECEO, while HS-GC-MS indicated a slightly reduced eugenol content (86.46%). The kneading method yielded the highest encapsulation efficiency for eugenol, whereas the co-precipitation–lyophilisation method was optimal for eucalyptol. DSC thermograms confirmed complex formation, and DLS analysis revealed nanostructures averaging 186.4 nm in diameter (PDI = 0.298). Antimicrobial assays showed MIC values ranging from 0.039 mg/mL to 10,000 mg/mL. Notably, ECEO and its *β*-CD complex displayed enhanced efficacy against *Escherichia coli* (0.039 mg/mL), surpassing the reference antibiotic gentamicin (0.049 mg/mL). **Conclusions:** *β*-Cyclodextrin encapsulation significantly enhances the stability and bioactivity of volatile antimicrobial compounds, thereby supporting their potential integration into advanced essential oil-based pharmaceutical formulations.

## 1. Introduction

The growing demand for natural compounds with proven biological activity has led to significant research into plant-derived bioactive substances in recent years. *Eugenia caryophyllata*, more commonly known as clove, is a plant of considerable interest due to its volatile oil, eugenol, which has been extensively studied for its antimicrobial, antioxidant, and anti-inflammatory properties [1,2].

Eugenol’s broad spectrum of biological activities has made it a promising candidate for use in pharmaceuticals, food preservation, and cosmetics. However, despite its potential, the practical application of eugenol is hindered by its inherent volatility, susceptibility to oxidation, and instability under environmental conditions, which limits its effectiveness as a therapeutic agent [3,4,5,6,7].

One promising solution to these challenges lies in the use of cyclodextrins (CDs), cyclic oligosaccharides that possess a hydrophilic outer surface and a hydrophobic inner cavity. Cyclodextrins have the ability to form inclusion complexes with various bioactive compounds, including terpenes such as eugenol [8,9,10]. When incorporated into cyclodextrins, volatile compounds are effectively encapsulated within the hydrophobic cavity, which provides protection from degradation, evaporation, and environmental factors such as heat and light. This encapsulation not only enhances the stability of the active ingredient but also offers the possibility of controlling its release, improving its solubility, and increasing its bioavailability. For these reasons, cyclodextrin-based systems have attracted considerable attention as effective carriers for bioactive molecules, especially for the stabilisation and controlled release of volatile compounds [11,12].

Although cyclodextrin inclusion complexes have shown promise in stabilising volatile oils, the methods used to prepare these complexes can significantly influence their encapsulation efficiency, stability, and overall performance. Traditional preparation techniques, such as solvent evaporation, co-precipitation, and spray-drying, have been widely employed to form cyclodextrin inclusion complexes with various active compounds. However, these methods often face limitations, including low encapsulation efficiencies, poor control over release profiles, and potential degradation of the encapsulated compounds during processing. Furthermore, while these methods have been effective in some instances, there remains a need for improved techniques that can more efficiently encapsulate volatile oils, such as eugenol, and preserve their biological activity [13,14]. In this regard, our study introduces a novel approach for preparing eugenol–*β*-cyclodextrin inclusion complexes using a co-precipitation–lyophilisation method, which, to the best of our knowledge, has not been previously explored in the literature. This research aims to investigate the potential of this combined method to improve the encapsulation of eugenol and enhance its antimicrobial properties. Co-precipitation followed by lyophilisation is expected to provide a more efficient way of encapsulating volatile compounds by stabilising them in a solid state, thus preventing the volatilisation and degradation that can occur during other processing methods. By comparing this new method with traditional techniques, such as the mortaring method, which has been widely used for complex preparation, we aim to identify the most effective approach for encapsulating eucalyptol, eugenol, and ECEO in cyclodextrins [15].

In addition, this study also explores the antimicrobial activity of eugenol–*β*-cyclodextrin, eucalyptol–*β*-cyclodextrin and ECEO–*β*-cyclodextrin complexes, as well as the antimicrobial activity of ECEO, eugenol, and eucalyptol. Eugenol’s antimicrobial activity has been well-documented; however, its efficacy is expected to increase when encapsulated in cyclodextrins. Previous research has shown that cyclodextrin inclusion complexes can improve the antibacterial potency of volatile oils, likely due to the controlled release of the active compound and protection from environmental factors. In particular, the ability of the complexes to prevent bacterial biofilm formation—an important factor in microbial resistance—will be evaluated as part of this study. Biofilm formation is a common mechanism by which bacteria resist antimicrobial agents, and any enhancement in the inhibition of biofilm development could have significant implications for the therapeutic use of eugenol [16,17].

Various characterisation techniques were employed to comprehensively understand the inclusion complexes of ECEO, eugenol, and eucalyptol with *β*-cyclodextrin. These methods include UV-Vis spectroscopy, gas chromatography–mass spectrometry (GC-MS), and headspace gas chromatography–mass spectrometry (HS-GC-MS) for determining entrapment efficiency and analysing the chemical composition of the complexes. The stoichiometry of the complexes was determined using Job’s method. Additionally, the structural properties of the complexes were investigated using Differential Scanning Calorimetry (DSC) and Scanning Electron Microscopy (SEM) to assess their morphology, thermal stability, and the nature of the inclusion.

This study is expected to make significant contributions to the field of cyclodextrin-based systems with bioactive compounds. By optimising the encapsulation of eugenol and enhancing its antibacterial activity, the findings of this research could lead to the development of more effective pharmaceutical formulations with improved stability, bioavailability, and antimicrobial efficacy. These findings indicate that this co-precipitation–lyophilisation approach could be further explored for the encapsulation of other volatile natural products with potential pharmaceutical applications.

## 2. Materials and Methods

### 2.1. Inclusion Complexes Preparation

#### 2.1.1. Reagents

All reagents used in the experiments were of analytical purity, purchased from the company Sigma-Aldrich (Merck KGaA; Darmstadt, Germany): eugenol (C_10_H_12_O_2_, CAS Number: 97-53-0, molecular weight: 164.2), eucalyptol (C_10_H_18_O, CAS Number: 470-82-6 molecular weight: 154.25), ethanol (C_2_H_6_O, CAS Number: 64-17-5, molecular weight: 46), methanol (CH_4_O, CAS Number: 67-56-1, molecular weight: 32), acetic acid (C_2_H_4_O_2_, CAS Number: 64-19-7, molecular weight: 60.05), *β*-Cyclodextrine (C_42_H_70_O_35_, CAS Number: 7585-39-9, molecular weight: 1134.98), and hexane (C_6_H_12_, CAS Number: 110-54-3, molecular weight: 84).

#### 2.1.2. Extraction of *Eugenia caryophyllata* Essential Oil

The dried *clove* inflorescences were purchased from Solaris Plant S.R.L. Volatile oil extraction was performed by steam water extraction using a standard Neo-Clevenger apparatus [18].

#### 2.1.3. Preparation of *β*-Cyclodextrin Inclusion Complexes

The encapsulation of *Eugenia caryophyllata* essential oil (eugenol and eucalyptol) in ***β***-cyclodextrin was carried out by four methods: kneading, co-precipitation, lyophilisation, and by a combined method of co-precipitation followed by lyophilisation.

##### Kneading Method

A total of 1.6 g *β*-cyclodextrin was mixed by kneading in a mortar with 280 μL of water until a paste was obtained 0.4 g of ECEO, respectively, 0.4 mg of eugenol and 0.4 mg of eucalyptol were added; the mixtures were continuously mixed for 15 min using a mortar and a pestle, then dried under vacuum [19].

##### Co-Precipitation Method

A total of 1.6 g *β*-cyclodextrin was dissolved in 14.4 mL of water:ethanol solution (3:1/v:v). The mixture was kept at 50 °C, under stirring, until the cyclodextrin was completely dissolved. ECEO/eugenol/eucalyptol (in 1 mL of ethanol) was added, keeping the *β*-cyclodextrin:compound (4:1/w:w) ratio. The mixtures were stirred for 4 h and dried under vacuum [20].

##### Lyophilisation Method

A total of 1.6 g *β*-cyclodextrin was dissolved in 14.4 mL of water:ethanol solution (3:1/v:v). ECEO/eugenol/eucalyptol (solubilized in 1 mL of ethanol) was added at room temperature, keeping the *β*-cyclodextrin:compound (4:1/w:w) ratio. The complexes were dried by lyophilisation [21].

##### Co-Precipitation Followed by Lyophilisation Method (Combined Method)

A total of 1.6 g *β*-cyclodextrin was dissolved in 14.4 mL of water:ethanol solution (3:1/v:v). The mixture was kept at 50 °C under stirring until the cyclodextrin was completely dissolved. ECEO/eugenol/eucalyptol (solubilized in 1 mL of ethanol) was added, keeping the *β*-cyclodextrin:compound (4:1/w:w) ratio. The mixtures were stirred for 4h and then lyophilized.

##### Lyophilisation Procedure

The samples were dried using a LaboGene ScanVac CoolSafe –11 °C 4 L Basic Freeze Dryer (LaboGene, Allerød, Denmark), which was operated at a temperature of –11 °C and under a reduced pressure of 0.02 mbar for 8 h.

### 2.2. Physicochemical Characterisation of Inclusion Complexes

#### 2.2.1. GC-MS Analysis

The chemical composition of ECEO and the entrapment efficiency of *β*-cyclodextrin complexes were performed using Thermo Scientific (Waltham, MA, USA) equipment with a Triplus autosampler. The GC-MS system consisted of a Focus GC gas chromatograph coupled with a Polaris Q ion hatch mass spectrometer and showed the following characteristics: the DB-5MS chromatographic capillary column type (l = 25 m; d = 0.25 mm; stationary-phase film thickness of 0.25 μm); an oven temperature programme including an initial temperature of 60 °C (3 min), followed by an increase of 10 °C/min to 200 °C (2 min), and then 12 °C/min to the final temperature of 240 °C; a carrier gas flow (helium) of 1 mL/min, with temperatures of the ion source and interface at 200 °C and 250 °C, respectively, and the detector operating in the mode with electron impact (70 eV). Detection was performed in the range of *m*/*z* 35–300, with the mass spectrometer operating in full-scan mode. The chromatograms were analysed using Xcalibur^TM^ 1.4 SR 1 software and the NIST 11 database.

For characterisation, the ECEO was diluted with hexane to a 1:10 volume ratio. In the case of *β*-cyclodextrin complexes, 50 mg of sample were mixed with 200 µL of water and 300 µL of dichloromethane. The obtained mixture was vortexed for 2 min, sonicated for 30 min, and centrifuged for 10 min at 10,000 rpm and 4 °C. The lower layer was collected. The procedure was repeated until a final volume of 1 mL was collected.

The headspace analysis was performed by placing 217 mg of each complex in a vial sealed with an aluminium cap with a silicone septum. ECEO (uncomplexed) was also analysed. Both the complexes and the samples were heated to 80 °C for 10 min. A volume of 500 µL of headspace gas was further analysed [22,23].

A standard solution of alkanes (C_8_–C_20_ in hexane) analysed under the same conditions as the samples was used to calculate the Kovats retention indices (IR). The calculation formula used for the Kovats retention index (IR) is presented below:

IR = 100n + 100 [(t_a_ − t_n_)/(t_n+1_ − t_n_)]

where

t = the retention time; a = the analyte of interest; n = the number of carbon atoms eluting before the peak of interest; and n + 1 = the number of carbon atoms eluting after the peak of interest.

The identification of the ECEO components was performed using the Kovats retention indexes obtained by calculation and compared to the NIST Chemistry WebBook database.

#### 2.2.2. Study of Host–Guest Equilibrium

##### Analysis of Stoichiometry of Inclusion Complexes

Both eugenol and ECEO are found in liquid form, with densities of 1.06 g/mL and 1.04 g/mL, respectively. A stock solution with a concentration of 1 mM of each species was prepared by weighing the necessary amount on an analytical balance and transferring it in a 50 mL volumetric flask, filled to the mark with ethanol as solvent. The working solution was obtained by diluting of a 1 mL stock solution with distilled water in a 25 mL volumetric flask and used to record UV spectra. The wavelength at which the chemical species have maximum of absorbance was identified, and for all determinations, 280 nm was considered optimal.

The mole ratio between each investigated species and *β*-CD has been established by Job’s method. For this purpose, a series of solutions was prepared, for which the mole fractions of eugenol and ECEO, respectively, varied between 0 and 1. The UV spectra were recorded using a Jasco V-530 (Tokyo, Japan) spectrometer. The plot of the absorbance difference ΔA (ΔA = absorbance difference in the chemical species without and with *β*-CD) multiplied by the mole fraction of the chemical species under investigation (R = [chemical species]/([chemical species] + [*β*-CD]) was plotted against R. The stoichiometry of the host–guest complex with *β*-CD was determined from the maximum of the Job plots [24].

##### Determination of Entrapment Efficiency

Entrapment efficiency was assessed by UV-Vis spectrometry using a Shimadzu (Kyoto, Japan) spectrophotometer, UV-1800, at λ = 275 nm, corresponding to the maximum absorption of eucalyptol for the eucalyptol–*β*-cyclodextrin complex and λ = 280 nm, corresponding to the maximum absorption of ECEO.

In total, 12.5 mg of the ECEO–*β*-cyclodextrin complex and the eugenol complex, respectively, were brought to 25 mL with absolute ethanol. The obtained suspensions were sonicated for 40 min and then centrifuged at 4800 rpm for 5 min. A total of 200 mg of the eucalyptol complex was mixed with 2 mL absolute ethanol. The obtained suspension was sonicated for 60 min and then centrifuged for 15 min at 4800 rpm [25].

#### 2.2.3. Differential Scanning Calorimetry (DSC) Analysis

DSC analysis was performed using a Perkin Elmer Diamond DSC (Waltham, MA, USA) under the following conditions: heating speed—controlled cooling from 10 °C/min; a temperature range of 0–300 °C; accuracy < ±0.1%; a sensitivity of 0.2 μW; and an inert argon atmosphere.

#### 2.2.4. Dynamic Light Scattering Analysis

The effective (hydrodynamic) diameter and particle size distribution were measured using a Beckman Coulter (Indiana, USA) Delsa^TM^Nano C instrument. A laser diode of 658 nm illuminated the nanoparticles, producing time-dependent fluctuations in the intensity of laser light, while the angle for size measurements was 165°. The measurements were performed at 25 °C. For data analysis, each sample measurement was performed in triplicate and the DelsaNano 3.73/2.30 software was used to further process the data.

#### 2.2.5. FTIR Characterisation

FTIR spectrum was acquired using the Tensor 27 spectrometer (Bruker Optics, Ettlingen, Germany) in the 4000–400 cm^–1^ spectral domain by averaging 64 scans with a resolution of 4 cm^–1^.

#### 2.2.6. Scanning Electron Microscopy

The surface morphology of the samples was obtained using an FEI Nova NanoSEM 630 scanning electron microscope (FEI Company, Hillsboro, OR, USA) with a TLD (Through-Lens-Detector) detector (FEI Company, Hillsboro, OR, USA), at an acceleration voltage of 5 kV and a working distance of 4.7 mm.

### 2.3. Antimicrobial Activity

Gram-negative strains (*Pseudomonas aeruginosa* ATCC 27853, *Escherichia coli* ATCC 25922, and *Enterobacter cloacae*—clinically isolated strain from skin and mucous infections) and Gram-positive strains (*Staphylococcus aureus* ATCC 25923, *Bacillus subtilis* ATCC 21332, and *Enterococcus faecalis* ATCC 49477) were analysed. All the strains tested were provided by the Microorganisms Collection of the Department of Microbiology, Faculty of Biology and Research Institute of the University of Bucharest.

#### 2.3.1. Qualitative Assay of the Antimicrobial Activity

Antimicrobial screening was performed by adapted disc diffusion using a microbial inoculum adjusted at 1.5 × 10^8^ CFU/mL (0.5 McFarland standard) from 24 h growth cultures on a nutrient agar medium. The plates were kept at room temperature for 15 min to ensure sample diffusion into the medium. The Petri dishes were incubated at 37 °C for 24 h. Antimicrobial activity was appreciated by measuring the growth inhibition zone diameters (GIZDs) [26].

#### 2.3.2. Quantitative Assay of the Antimicrobial Activity

The minimum inhibitory concentration (MIC) was determined using a modified binary serial microdilution assay in liquid media with 96-well microtiter plates. For each sample, two-fold serial dilutions were prepared in 150 µL of the appropriate broth medium containing the standard inoculum. The microtiter plates were then incubated at 37 °C for 24 h. MIC values were established through visual inspection and spectrophotometric analysis, measuring absorbance at 620 nm using the BIOTEK SYNERGY-HTX ELISA Multi-Mode Reader (Winooski, VT, USA) [27].

#### 2.3.3. Semiquantitative Assessment of the Microbial Adherence to the Inert Substratum

Biofilm formation on the inert substratum (MBEC) was evaluated using the same two-fold serial microdilution technique. After 24 h of incubation, the 96-well plates used to determine the minimum inhibitory concentration were washed with sterile physiological water to remove the medium, compound residues, and non-adherent microorganisms and then dried. Over the adhered bacteria, 99.8% methanol was added and kept at room temperature for 15 min. After removing the methanol, the biofilms were stained with 1% crystal violet for 10 min, washed with sterile water, and dried at room temperature. The stained biofilm was resuspended with 33% acetic acid, and the absorbance was measured at 490 nm [26,27].

### 2.4. Statistical Analysis

The data results were statistically analysed with GraphPad Prism 10.3 from GraphPad Software, San Diego, CA (USA). All experiments were performed in three independent determinations. The results are expressed as means ± SDs (standard deviations), analysed using a one-way analysis of variance followed by a multiple comparison test, chosen according to the experimental design. The differences between groups/samples were considered statistically significant when the *p*-value was <0.05.

## 3. Results

### 3.1. Evaluation of Physicochemical Characterisation of Inclusion Complexes

#### 3.1.1. GC-MS and HS-GC-MS

The ECEO concentration (a pale yellow oil with a characteristic odour) was 3.96% (*v*/*w*) (3.96 mL of volatile oil per 100 g of inflorescences) and stored at 4 ± 2 °C during the study.

The ECEO chemical composition, as well as the compounds identified in the *β*-cyclodextrin-encapsulated ECEO by the GC-MS and HS-GC-MS methods, are presented in Table 1 and Table 2, respectively.

The chemical structures of the most abundant constituents identified by GC-MS and HS-GC-MS are presented in Table 3 (eugenol, eucalyptol, (E)-*β*-caryophyllene, and eugenyl acetate).

#### 3.1.2. Stoichiometry of Inclusion Complexes

**Figure 1:**
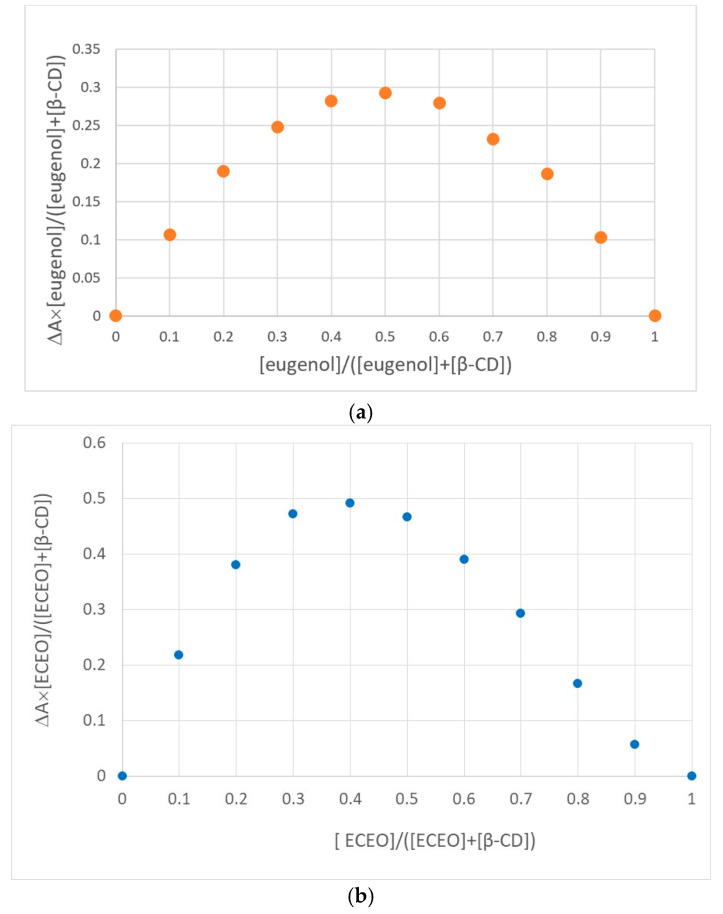
(**a**) Job’s plot for eugenol–*β*-CD inclusion complex. (**b**) Job’s plot for ECEO–*β*-CD inclusion complex.

As is well-known, *β*-CD has no absorption in the UV-VIS region of the electromagnetic spectrum. Therefore, to study the host–guest equilibrium between *β*-CD on the one hand and eugenol and ECEO on the other hand, a study on the UV-VIS behaviour of these species was carried out. Thus, UV-VIS spectra of 1 mM solutions of eugenol and ECEO, respectively, were recorded. Both spectra have a maximum absorption band at 280 nm, which allowed us to choose this wavelength for monitoring *β*-CD complex formation. Thus, the stoichiometry of the formed complexes was obtained by applying the continuous variation method known as Job’s method, adapted for these equilibria involving cyclodextrins [28]. The Job plots for the eugenol–*β*-CD and ECEO–*β*-CD systems are shown in Figure 1a and 1b, respectively. The shape of the curve in Figure 1a indicates 1:1 stoichiometry between the host and guest molecules, since the maximum of the graph is located at a value of 0.5 for the eugenol mole fraction. On the contrary, the equilibrium between ECEO and *β*-CD shows a stoichiometry of 1:2, with the maximum of Job’s plot being at 0.33 for the essential oil mole fraction. This can be explained by the complexity of the ECEO composition, as it also contains, in addition to eugenol, other chemical species that can be hosted by two molecules of *β*-CD simultaneously, hence displaying different stoichiometry than with eugenol alone.

#### 3.1.3. Entrapment Efficiency

The results obtained for encapsulation efficiency are presented in Table 4. The encapsulation efficiencies (EE%) of eucalyptol, eugenol, and *Eugenia caryophyllata* essential oil (ECEO) were evaluated using four different complexation methods: kneading (K), co-precipitation (C), lyophilisation (L), and a combined co-precipitation–lyophilisation method (C–L). For eucalyptol, the highest encapsulation efficiency was achieved by the C–L method (95.62 ± 3.7%), followed closely by kneading (90.06 ± 3.3%). The co-precipitation method resulted in a significantly lower efficiency (48.68 ± 2.8%). In the case of eugenol, the kneading method provided the best encapsulation efficiency at 99.38 ± 5.2%, followed by lyophilisation (77.66 ± 4.9%). The co-precipitation and C–L methods yielded considerably lower efficiencies of 55.01 ± 4.7% and 53.96 ± 4.4%, respectively. For ECEO, kneading again achieved the highest efficiency at 99.40 ± 1.9%, while lyophilisation reached 79.02 ± 2.0%. Co-precipitation (69.26 ± 2.4%) and C–L (60.48 ± 1.9%) methods resulted in lower encapsulation efficiencies.

Overall, the kneading method proved to be the most effective for encapsulating both eugenol and ECEO, while the combined co-precipitation–lyophilisation technique exhibited the highest performance for eucalyptol.

#### 3.1.4. Differential Scanning Calorimetry

The DSC analysis of *β*-cyclodextrin revealed a broad endothermic peak in the temperature range of 75–125 °C, characteristic of the loss of hydration water from the cyclodextrin molecule. For eucalyptol, the DSC curve displayed a broad peak between 116 and 130 °C, with two maxima at 116.72 °C and 125.27 °C, which are lower than the values reported in the literature (176 °C). Eugenol exhibited two distinct endothermic peaks, one at 127.7 °C and another at 232.7 °C. The peak at 127.7 °C aligns with findings from a 2022 study, indicating thermal oxidation of eugenol, while the peak at 232.7 °C corresponds to its boiling point [29].

**Figure 2:**
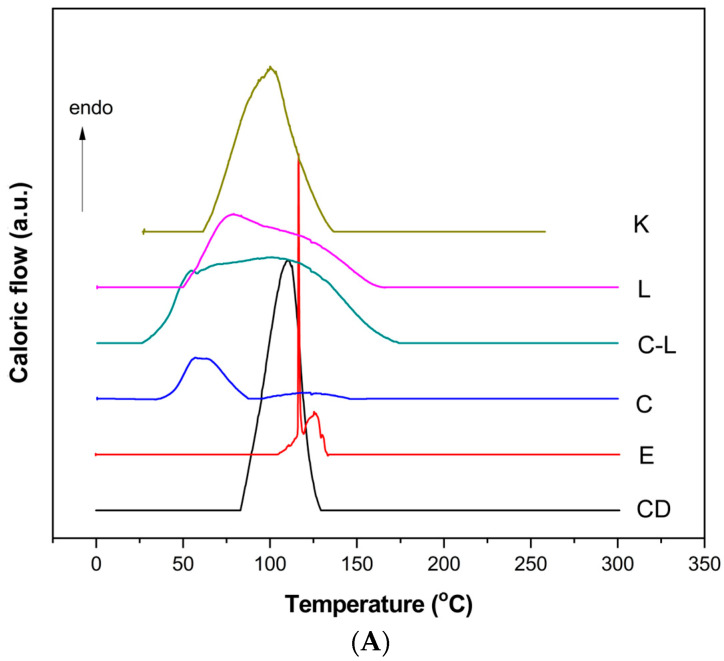

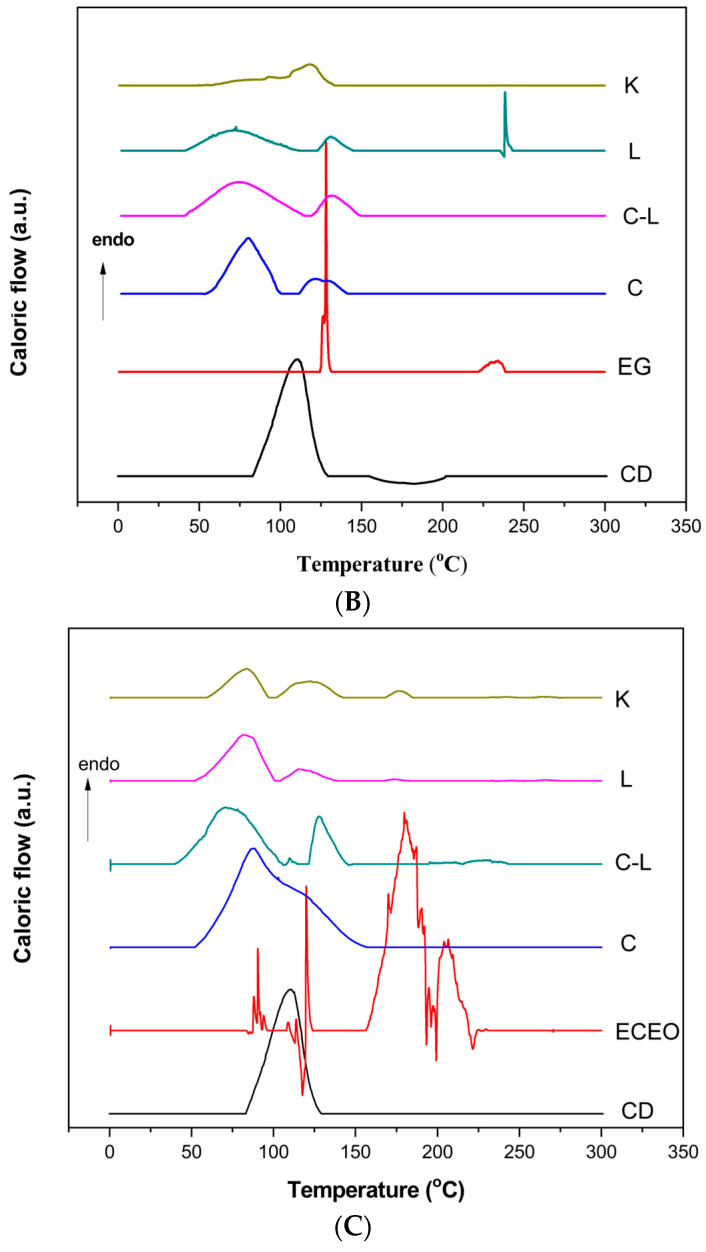
Differential Scanning Calorimetry curves of the eucalyptol–*β*-cyclodextrin (**A**), eugenol–*β*-cyclodextrin (**B**), and ECEO–*β*-cyclodextrin (**C**) complexes. CD: *β*-cyclodextrin; E: eucalyptol; EG: eugenol; K: encapsulation by kneading; L: encapsulation by lyophilisation; C-L: encapsulation by co-precipitation–lyophilisation; C: encapsulation by co-precipitation; ECEO: *Eugenia caryophyllata* essential oil.

The DSC curves obtained for the eucalyptol–*β*-cyclodextrin, eugenol–*β*-cyclodextrin, and ECEO–*β*-cyclodextrin complexes are shown in Figure 2A–C.

#### 3.1.5. SEM Analysis of the Inclusion Complexes

**Figure 3:**
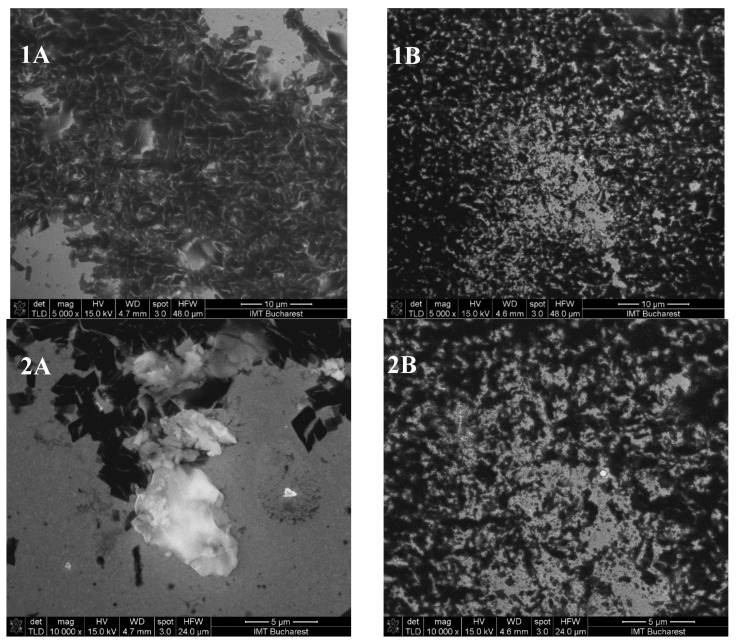
SEM images of eucalyptol–*β*-cyclodextrin complexes obtained by lyophilisation (**A**) and kneading (**B**) at magnifications of ×5000 (**1**) and ×10,000 (**2**).

**Figure 4:**
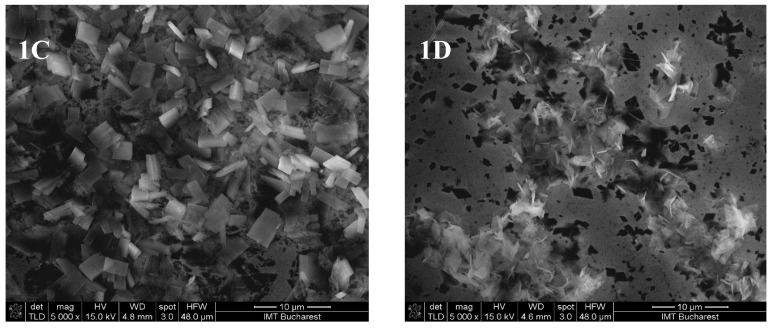

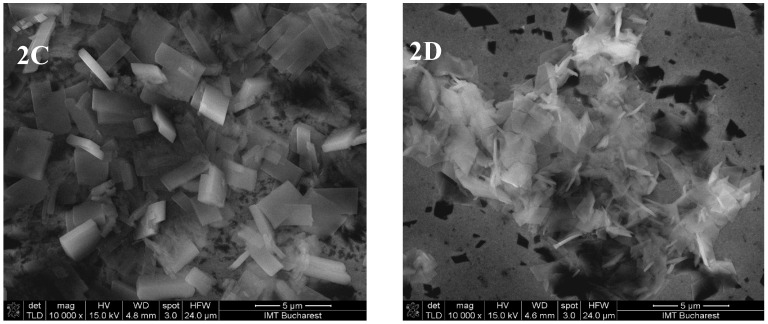
SEM images of eugenol–*β*-cyclodextrin complexes obtained by lyophilisation (**C**) and kneading (**D**) at magnifications of ×5000 (**1**) and ×10,000 (**2**).

**Figure 5:**
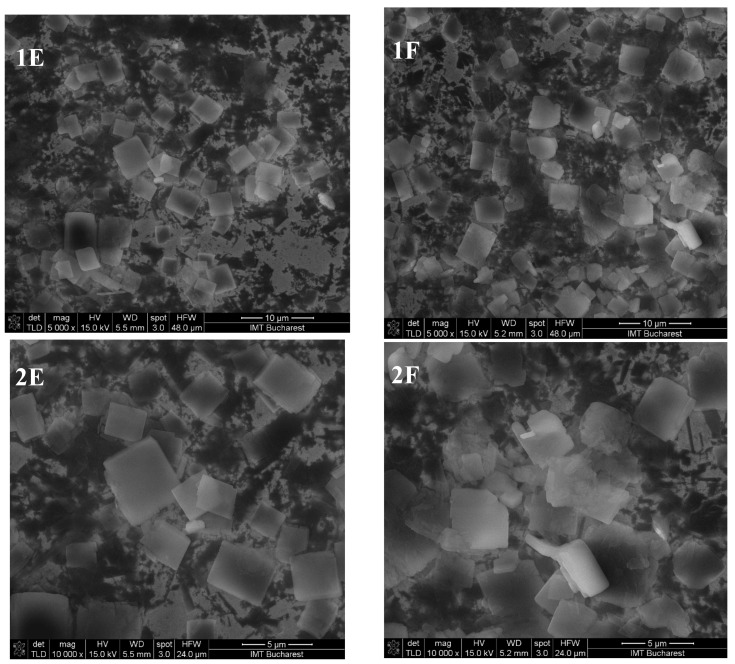
SEM images of ECEO–*β*-cyclodextrin complexes obtained by lyophilisation (**E**) and kneading (**F**) at magnifications of ×5000 (**1**) and ×10,000 (**2**).

SEM images of the complexes formed by kneading and lyophilisation for eucalyptol, eugenol, and ECEO at magnifications of ×5000 and ×10,000 are shown in Figure 3, Figure 4 and Figure 5.

The *β*-cyclodextrin complexes with eugenol exhibit parallelepiped particle morphology, distinct from the complexes with ECEO, while the eucalyptol complexes show the formation of conglomerates.

#### 3.1.6. Particles Size Distribution

Particle size distribution analysis was analysed for the ECEO–*β*-cyclodextrin complex and the (E)-*β*-cyclodextrin complex obtained via the kneading method.

Dynamic light scattering analysis of the ECEO–*β*-cyclodextrin complex revealed nanostructured assemblies with a hydrodynamic diameter of 186.4 nm and a polydispersity index (PI) of 0.298. The diffusion coefficient was recorded at 2.127 × 10^−8^ cm^2^/s, while the estimated molecular weight was 2.209 × 10^9^ g/mol.

**Figure 6:**
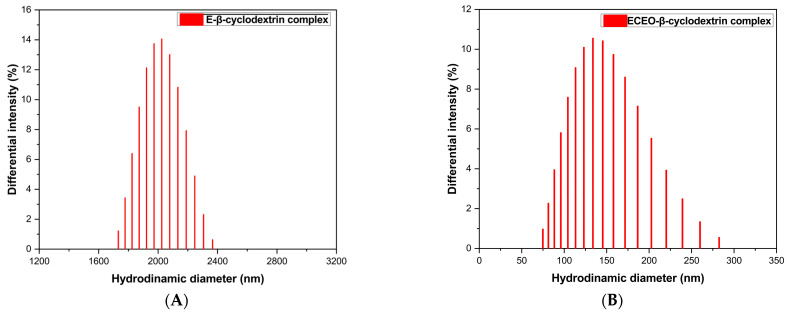
Particle size distribution of the eugenol–*β*-cyclodextrin (**A**) and ECEO–*β*-cyclodextrin (**B**) complexes via the kneading method. ECEO: *Eugenia caryophyllata* essential oil.

For the *β*-cyclodextrin–eucalyptol complex, DLS analysis indicated a markedly larger hydrodynamic diameter of 2929.0 nm. The system exhibited a PI value of –2.651, falling outside the accepted range for DLS interpretation. The diffusion coefficient was notably lower, at 1.354 × 10^−9^ cm^2^/s. The particle size distribution of the eugenol–*β*-cyclodextrin and ECEO–*β*-cyclodextrin complexes via the kneading method are presented in Figure 6.

These observations were corroborated by Scanning Electron Microscopy (SEM) analysis, which revealed discrete, well-defined particles for the clove oil complex and significant aggregation for the eucalyptol complex.

#### 3.1.7. FTIR Analysis Results

FTIR (Fourier Transform Infrared Spectroscopy) analysis was conducted on the ECEO–*β*-cyclodextrin, E–*β*-cyclodextrin, and EG–*β*-cyclodextrin complexes prepared using the kneading method, as it was considered the most representative sample. FTIR spectra of the ECEO–*β*-cyclodextrin complex illustrated the specific bands of the functional groups, corresponding to the eugenol molecule as the main component of ECEO. The IR spectra are presented in Figures S1–S3 in the Supplementary Material of this paper.

Infrared spectroscopic investigation revealed distinct modifications indicative of inclusion complex formation between eugenol and *β*-cyclodextrin. The broad O–H stretching vibration of *β*-cyclodextrin, initially detected at 3304 cm^−1^, exhibited a slight shift to 3300 cm^−1^ in the complex, reflecting alterations in hydrogen bonding interactions upon guest encapsulation. The aromatic =C–H stretching band of eugenol at 3080 cm^−1^ decreased in intensity or overlapped within the complex spectrum, suggesting the partial accommodation of the aromatic ring within the *β*-cyclodextrin cavity. Moreover, the C=C stretching signals of eugenol at 1637 and 1510 cm^−1^ experienced minor displacements to 1639 and 1512 cm^−1^, respectively, indicating the preservation of the aromatic framework alongside new host–guest interactions. A notable shift in C–O phenolic stretching from 1265 to 1282 cm^−1^ further evidenced the involvement of hydroxyl groups in hydrogen bond formation with the cyclodextrin host. The glycosidic C–O–C absorptions of *β*-cyclodextrin (1150–1020 cm^−1^) maintained their position, although with subtle intensity variations post complexation, confirming the structural integrity of the carrier [30,31,32,33].

For eucalyptol, IR analysis displayed characteristic absorptions for C–H stretching at 2968, 2920, and 2879 cm^−1^, CH_2_ and CH_3_ bending vibrations at 1465, 1446, 1375, and 1359 cm^−1^, and C–O–C asymmetric stretches at 1234 and 1215 cm^−1^. Upon complexation with *β*-cyclodextrin, the broad O–H band at 3301 cm^−1^ narrowed and weakened, indicating hydrogen bond formation and the displacement of water from the cavity. Additionally, observable shifts in the C–H region and adjustments within the fingerprint area, notably at 1371, 1157, 1080, and 1024 cm^−1^, confirmed the successful molecular inclusion of eucalyptol. The disappearance or attenuation of specific eucalyptol-related absorptions and the appearance of new features further substantiated complex formation, consistent with reported behaviour of cyclodextrin-based systems [33,34].

Similarly, the clove essential oil (ECEO) spectrum showed typical O–H stretching at 3522 and 3440 cm^−1^, alongside C–H stretching vibrations for aromatic and aliphatic groups at 3080, 2940, and 2837 cm^−1^. A distinct peak at 1759 cm^−1^ corresponded to ester carbonyl stretching, while absorptions at 1637, 1602, and 1512 cm^−1^ were associated with aromatic C=C vibrations. In the fingerprint region, multiple bands including 1463, 1421, 1367, 1265, 1230, and 1031 cm^−1^ were ascribed to CH bending and C–O deformations. After complexation with *β*-cyclodextrin, the complex exhibited a broad O–H stretch at 3304 cm^−1^, alongside a decrease in intensity and slight shift for C–H stretches, now observed at 2923 cm^−1^. Notably, the ester carbonyl band at 1759 cm^−1^ was significantly reduced or absent, with concomitant shifts in the aromatic region (1634 and 1516 cm^−1^) and alterations in the fingerprint domain (1369, 1271, 1151, 1080, and 1026 cm^−1^) [30,31,33].

### 3.2. Analysis of Antimicrobial Activity

#### 3.2.1. Qualitative Assessment of the Antimicrobial Activity

Antimicrobial activity was qualitatively evaluated by determining the growth inhibition zone diameters (GIZDs) that appeared around the samples. The results obtained are presented in Table S1 of the Supplementary Material. The qualitative evaluation of antimicrobial activity (Table S1) showed that the most pronounced effects were observed, as expected, with ECEO and its *β*-cyclodextrin complex, followed by eugenol and the eugenol–*β*-cyclodextrin complex, against both Gram-positive and Gram-negative bacterial strains. In contrast, eucalyptol did not exhibit inhibitory activity against Gram-positive bacterial strains, but it did show growth inhibitory effects on Gram-negative strains.

#### 3.2.2. Quantitative Assessment of the Antimicrobial Activity

**Figure 7:**
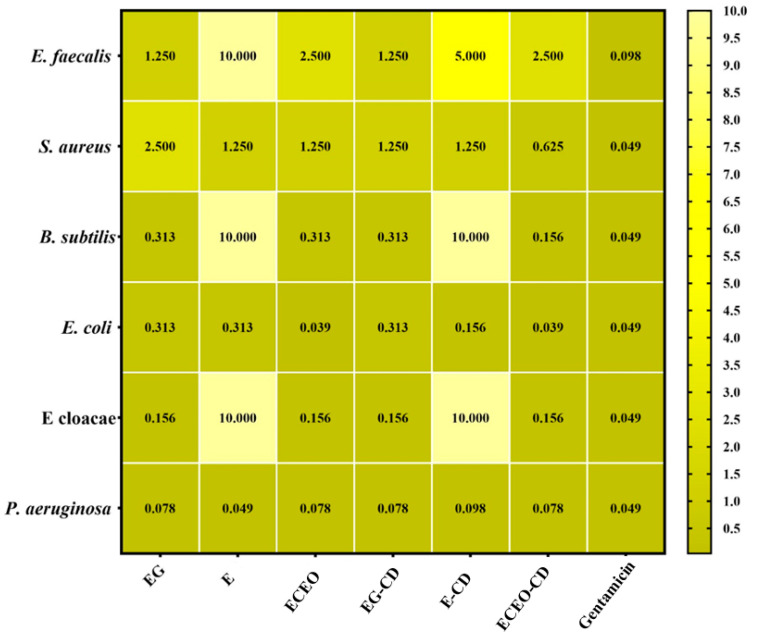
Heatmap of bioactive components and complexes’ MIC values (mg/mL). Legend: Eugenol: EG; Eucalyptol: E; *Eugenia caryophyllata* essential oil: ECEO; *β*-cyclodextrin complex with EG: EG-CD; *β*-cyclodextrin complex with E: E-CD; *β*-cyclodextrin complex with ECEO: ECEO-CD. The scale bar displays variations in the sensitivity of the strains from the highest (green) to the lowest (cream). The influence of the bioactive compounds on each microbial strain was statistically analysed using one-way ANOVA and Tukey’s multiple comparisons test. The data results were statistically significant (*p* < 0.05). *E. faecalis*—*Enterococcus faecalis*; *S. aureus*—*Staphylococcus aureus*; *B. subtilis*—*Bacillus subtilis*; *E. coli*—*Escherichia coli*; *E. cloacae*—*Enterobacter cloacae*; *P. aeruginosa*—*Pseudomonas aeruginosa*.

The quantitative evaluation of antimicrobial activity entails determining the minimum inhibitory concentration (MIC), which is the lowest concentration of an antimicrobial agent required to inhibit the visible growth of a microorganism. The MIC results are presented in Figure 7.

The MIC values ranged from 0.039 mg/mL to 10,000 mg/mL. The lowest MIC values, indicating the highest antimicrobial efficacy, were observed for clove oil and its *β*-cyclodextrin inclusion complex, both exhibiting an MIC of 0.039 mg/mL against the *Escherichia coli* strain. These findings suggest superior antimicrobial activity compared to gentamicin, for which a MIC value of 0.049 mg/mL was recorded. Among the bacterial strains tested, *Pseudomonas aeruginosa* exhibited the highest sensitivity to clove oil, eugenol, and eucalyptol, as well as to their respective inclusion complexes. The highest MIC values were recorded for eucalyptol and its inclusion complex for *Bacillus subtilis* (10 mg/mL for both samples), *Enterobacter cloacae* (10 mg/mL for both samples), and *Enterococcus faecalis* strains (10 mg/mL and 5 mg/mL, respectively) strains. Regarding the antimicrobial activity of free bioactive compounds compared to those encapsulated in *β*-cyclodextrin, the results were generally similar, with encapsulated compounds demonstrating superior efficacy in some cases. This enhanced activity may be attributed to the ability of cyclodextrin to encapsulate volatile compounds, enabling their gradual release and thus providing prolonged antimicrobial protection compared to the free compounds.

#### 3.2.3. Semiquantitative Assessment of the Microbial Adherence to the Inert Substratum

**Figure 8:**
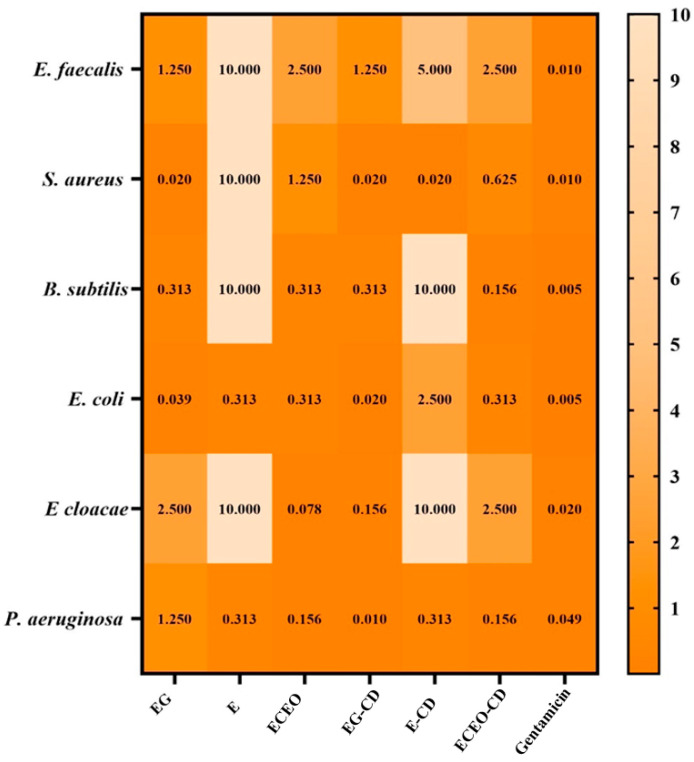
Heatmap of bioactive components and complexes’ MBEC values (mg/mL). Legend: Eugenol: EG; Eucalyptol: E; *Eugenia caryophyllata* essential oil: ECEO; *β*-Cyclodextrin complex with EG: EG-CD; *β*-cyclodextrin complex with E: E-CD; *β*-Cyclodextrin complex with ECEO: ECEO-CD. The scale bar displays variations in the sensitivity of the strains from the highest (orange) to the lowest (peach pink). The influence of the bioactive compounds on each microbial strain was statistically analysed using one-way ANOVA and Tukey’s multiple comparisons test. The data results were statistically significant (*p* < 0.05). *E. faecalis*—*Enterococcus faecalis*; *S. aureus*—*Staphylococcus aureus*; *B. subtilis*—*Bacillus subtilis*; *E. coli*—*Escherichia coli*; *E. cloacae*—*Enterobacter cloacae*; *P. aeruginosa*—*Pseudomonas aeruginosa*.

The semiquantitative evaluation of microbial adherence to inert surfaces focused on assessing the capacity of microorganisms to attach and develop biofilms on different surfaces, thus the minimum biofilm eradication concentration (MBEC) is a key parameter for assessing the efficacy of antimicrobial agents. The MBEC results obtained in this study are presented in Figure 8.

The MBEC values ranged from 0.010 mg/mL to 10 mg/mL, with the lowest concentration recorded for the eugenol–*β*-cyclodextrin complex against the *Pseudomonas aeruginosa* strain. This value was even lower than that obtained for gentamicin. Overall, the encapsulated compounds exhibited superior biofilm eradication efficacy compared to their free counterparts. As expected, the least effective compound in eliminating bacterial biofilms was eucalyptol and its complex, with MBEC values reaching up to 10 mg/mL.

## 4. Discussion

GC-MS analysis (Table 1) showed that the most abundant constituent of the ECEO is eugenol (90.67%), followed by eugenyl acetate (4.77%) and (E)-*β*-caryophyllene (3.98%). Similar results were obtained following the HS-GC-MS analysis, noting that eugenol was identified in a lower percentage (86.46%). The literature provides different eugenol concentrations in the ECEO composition, between 47.67% and 88.58% [35,36].

The chemical composition of the inclusion complexes of ECEO with *β*-cyclodextrin using various methods (kneading, co-precipitation, lyophilisation, and co-precipitation–lyophilisation) were compared (Table 2). It was observed that eugenol was encapsulated in the highest proportion across all the preparation methods. This outcome was anticipated, as GC-MS analysis revealed eugenol as the major constituent of the essential oil. While the inner cavity of *β*-cyclodextrin is predominantly hydrophobic, the inclusion of essential oil constituents with amphiphilic character, such as eugenyl acetate, eucalyptol, and p-cymene, is facilitated by their dimensional compatibility with the cavity and the ability of polar functional groups to interact with the hydrophilic rims of cyclodextrin molecules.

As reported in the literature, complexation efficiency is influenced by molecular size, shape, and the distribution of hydrophobic and polar domains within guest molecules. The encapsulation of slightly polar volatiles is made possible when the hydrophobic regions reside within the cavity, while polar groups remain exposed at the cavity entrances, stabilising the inclusion complex through hydrogen bonding or dipole interactions [30,31,34].

Encapsulation efficiency calculations indicated that the kneading method provided the highest complexation efficiency, followed by lyophilisation, with co-precipitation being the least efficient.

The highest encapsulation efficiency of ECEO in *β*-cyclodextrin was achieved using the kneading method (Table 4), followed by lyophilisation, while the co-precipitation combined with lyophilisation method showed the lowest efficiency. A similar trend was observed for pure eugenol encapsulation. In contrast, the co-precipitation–lyophilisation combined technique demonstrated the highest complexation efficiency for eucalyptol, followed by kneading and co-precipitation.

The superior encapsulation efficiency of eugenol obtained by the kneading method can be attributed to the specific features of the preparation process, which favour intimate contact between *β*-cyclodextrin and the guest molecule. The gradual addition of a minimal amount of solvent during kneading promotes localised supersaturation around the cyclodextrin particles, enhancing host–guest interactions and facilitating the effective inclusion of eugenol within the hydrophobic cavity. Furthermore, the mechanical energy applied during kneading increases molecular mobility and alignment, improving the probability of complex formation. Unlike other methods, kneading is performed under mild conditions, which minimises the risk of volatilisation or degradation of volatile compounds, like eugenol, contributing to higher retention and encapsulation efficiency. Additionally, the process allows the hydrophobic aromatic moiety of eugenol to be accommodated within the cavity while the phenolic hydroxyl group may remain partially exposed at the rim of the cyclodextrin, stabilising the inclusion complex through hydrogen bonding interactions. These factors collectively explain the superior encapsulation performance achieved via the kneading method, consistent with findings reported in similar studies on essential oil complexation [30,34].

Studies by UV-VIS spectrometry confirmed the formation of host–guest complexes between *β*-CD and either eugenol or ECEO, with absorption maxima at 280 nm. Job’s method revealed 1:1 stoichiometry for the eugenol–*β*-CD complex and 1:2 stoichiometry for the ECEO–*β*-CD complex, likely due to the heterogeneous composition of the essential oil, which allows simultaneous interactions with two *β*-CD molecules.

In a 2017 study, the effect of the ECEO–*β*-cyclodextrin ratio (m/m) on encapsulation efficiency was evaluated. Complexes were prepared via co-precipitation at various ratios, with the highest encapsulation efficiency of 63.63% recorded at a ratio of 4:96. Additionally, the literature reports the preparation of *β*-cyclodextrin complexes with ECEO and eugenol via lyophilisation at a 1:1 ratio, yielding encapsulation efficiencies of 90.15% for eugenol complexes and 77.74% for ECEO complexes [37].

The DSC analysis showed, for eucalyptol and eugenol complexes prepared by lyophilisation, kneading, co-precipitation, and the combined method, two overlapping peaks: one corresponding to dehydration, which occurred at lower temperatures compared to pure *β*-cyclodextrin, and the other related to vaporisation. These shifts indicate that the compounds had entered the cyclodextrin cavity, replacing some of the hydration water. In the ECEO complexes, regardless of the preparation method, a broad peak in the 50–125 °C range was observed, corresponding to the loss of cyclodextrin hydration water, followed by a peak associated with eugenol.

In a study by Ma et al. (2018), DSC thermograms of ECEO inclusion complexes with *β*-cyclodextrin exhibited broad endothermic peaks in the temperature range of 50–125 °C, attributed to the dehydration of cyclodextrin molecules. Subsequent peaks were associated with the thermal events of eugenol, the primary constituent of ECEO [38].

Additionally, research by Adjali et al. (2022) on hydroxypropyl–*β*-cyclodextrin (HP–*β*-CD) inclusion complexes with ECEO identified endothermic peaks around 179 °C in lyophilized and kneaded samples. These peaks were postulated to correspond to the vaporisation temperatures of minor components such as p-cymene and limonene, based on the existing literature [25].

The SEM images (Figure 3, Figure 4 and Figure 5) confirm that the morphology of *β*-cyclodextrin undergoes significant alterations after complexation, consistent with previous literature reports [39]. However, no substantial differences in particle shape were observed between the different preparation methods. The *β*-cyclodextrin–clove oil complex exhibited relatively well-defined, discrete particles with a moderately uniform size distribution and smooth surface morphology. These observations are consistent with the DLS results, which indicate a moderately monodisperse and stable system.

Similarly, the *β*-cyclodextrin–eugenol complex displayed small, slightly irregular particles, with minimal evidence of aggregation in the SEM images.

On the other hand, the SEM images of the *β*-cyclodextrin–eucalyptol complex revealed the presence of irregular, aggregated structures, with particle assemblies significantly larger than those typically reported for inclusion complexes. These morphological findings corroborate the DLS measurements that are further presented, where an unusually high hydrodynamic diameter and an abnormal polydispersity index were recorded, indicative of system instability and supramolecular aggregation [40].

The DLS analysis of the ECEO–*β*-cyclodextrin complex revealed the formation of nanostructured assemblies with a hydrodynamic diameter of 186.4 nm, suggesting that the encapsulation process results in well-defined nanoparticles. The polydispersity index (PI) of 0.298 indicates a moderately monodisperse system, reflecting a relatively uniform particle size distribution, which is desirable for pharmaceutical and delivery applications [41,42].

The diffusion coefficient (2.127 × 10^−8^ cm^2^/s) confirms the presence of larger molecular aggregates, consistent with the encapsulation of ECEO within the cyclodextrin cavity and a possible supramolecular self-assembly. Furthermore, the estimated molecular weight (2.209 × 10^9^ g/mol) supports the hypothesis of high-molecular-weight complexes, likely involving multiple *β*-cyclodextrin units interacting with the encapsulated essential oil molecules [30].

On the other hand, the DLS analysis of the *β*-cyclodextrin–eucalyptol inclusion complex revealed the formation of micro- and nanostructured assemblies with a notably larger hydrodynamic diameter of 2929.0 nm. This value suggests the aggregation of multiple *β*-cyclodextrin units associated with eucalyptol molecules, leading to the formation of supramolecular structures of micrometric size, substantially exceeding those recorded for the *β*-cyclodextrin–clove oil system [43].

Interestingly, the reported polydispersity index (PI) of –2.651 falls outside the valid interpretation range for DLS measurements, as PI values should typically range between 0 (perfectly monodisperse) and 1 (highly polydisperse). This abnormal reading may indicate system instability, likely resulting from the formation of large, unstable aggregates. SEM analysis also confirmed the formation of aggregates [40].

The diffusion coefficient of 1.354 × 10^−9^ cm^2^/s further confirms the presence of slow-moving, large molecular assemblies, consistent with the formation of high-molecular-weight aggregates and possible interparticle interactions. These findings suggest that, under the tested conditions, the *β*-cyclodextrin–eucalyptol system tends to self-associate into larger, less uniform supramolecular structures, which may impact their pharmaceutical applicability unless formulation parameters are optimised to reduce aggregation and improve size distribution [30].

Overall, while complex formation is evident, the relatively large particle size and anomalous PI indicate the need for process refinement to obtain stable, pharmaceutically acceptable nanostructures with controlled size and distribution.

The FTIR analysis confirms the successful inclusion of ECEO, EG, and E into the cyclodextrin cavity, stabilised by hydrogen bonding and hydrophobic effects. The cumulative spectral changes confirm the encapsulation of ECEO constituents, particularly eugenol, within the hydrophobic cavity of *β*-cyclodextrin, contributing to the stabilisation of the volatile components’ bioactivity [33,44].

This study investigates the antimicrobial activity of ECEO, eugenol, and eucalyptol, along with their inclusion complexes with *β*-cyclodextrin, against various bacterial strains. The MIC values ranged from 0.039 mg/mL to 10.000 mg/mL. The lowest MIC values, indicating the highest antimicrobial efficacy, were observed for ECEO and its *β*-cyclodextrin inclusion complex, both exhibiting a MIC of 0.039 mg/mL against the *Escherichia coli* strain. These findings suggest superior antimicrobial activity compared to gentamicin, for which a MIC value of 0.049 mg/mL was recorded. Among the bacterial strains tested, *Pseudomonas aeruginosa* exhibited the highest sensitivity to ECEO, eugenol, and eucalyptol, as well as to their respective inclusion complexes. The highest MIC values were recorded for eucalyptol and its inclusion complex, with MICs of 10 mg/mL for *Bacillus subtilis* and *Enterobacter cloacae* strains, and 10 mg/mL and 5 mg/mL, respectively, for the *Enterococcus faecalis* strain. The antimicrobial activity of free bioactive compounds compared to those encapsulated in *β*-cyclodextrin was generally similar, with encapsulated compounds demonstrating superior efficacy in some cases. This enhanced activity may be attributed to the ability of cyclodextrin to encapsulate volatile compounds, enabling their gradual release and thus providing prolonged antimicrobial protection compared to the free compounds.

The MBEC values ranged from 0.010 mg/mL to 10 mg/mL, with the lowest concentration recorded for the eugenol–*β*-cyclodextrin complex against the *Pseudomonas aeruginosa* strain, the value also lower than that obtained for gentamicin. This value was even lower than that obtained for gentamicin. Overall, the encapsulated compounds exhibited superior biofilm eradication efficacy compared to their free counterparts. As expected, eucalyptol and its complex were the least effective samples in eliminating bacterial biofilms, with MBEC values reaching up to 10 mg/mL.

These findings highlight the potential of essential oil-based inclusion complexes as effective antimicrobial agents, with possible applications in food preservation, pharmaceuticals, and healthcare.

The results indicated enhanced antibacterial activity following the encapsulation of ECEO and eugenol in *β*-cyclodextrin, likely due to improved stability. The antimicrobial properties of the volatile oil and its complexes are predominantly attributed to eugenol, which is well-known for its antibacterial and antifungal effects. Eugenol has demonstrated remarkable antimicrobial activity against a wide range of Gram-positive and Gram-negative bacteria, as well as certain fungi [45].

For instance, eugenol has shown significant bactericidal activity against *Escherichia coli*, with a MIC of 0.125 μg/mL and a MBEC of 0.250 μg/mL. Time–kill curves revealed a rapid reduction in *Escherichia coli* to undetectable levels in the presence of eugenol, suggesting membrane permeability alteration and intracellular content leakage [46].

Studies indicate that eugenol exerts its antimicrobial effect by interacting with membrane proteins, inhibiting essential enzymatic functions, and disrupting the cytoplasmic membrane of microorganisms. This disruption compromises membrane integrity, increases permeability, and leads to the leakage of essential intracellular components, ultimately resulting in bacterial cell death [45,47].

Additionally, eugenol has demonstrated the ability to inhibit bacterial biofilm formation and reduce the expression of virulence factors in various bacterial species, including *Staphylococcus aureus* and *Escherichia coli*. These properties highlight the potential of eugenol as a natural antimicrobial agent in various applications, including the food and medical industries [48].

Research indicates that ECEO is effective against pathogens such as *Escherichia coli*, *Staphylococcus aureus*, and *Pseudomonas aeruginosa*. In a study assessing its efficacy, a 0.4% concentration of ECEO at 21 °C reduced the bacterial population by five logarithmic orders, demonstrating its potent bactericidal activity. However, the presence of organic matter can diminish its effectiveness, though not entirely negate it [49]. Additionally, ECEO has demonstrated efficacy against antibiotic-resistant bacteria. For instance, it has been shown to inhibit methicillin-resistant *Staphylococcus aureus* (MRSA) at concentrations of 20 µg/mL and 40 µg/mL, indicating its potential as an alternative or complementary treatment option in combating resistant strains [50]. Other studies evaluated ECEO antibacterial activity against *Propionibacterium acnes*, a bacterium associated with acne. The findings revealed significant antibacterial effects, suggesting potential therapeutic applications in dermatology [51].

ECEO stability and efficacy are limited; therefore, its encapsulation using *β*-cyclodextrin (*β*-CD) has been explored. *β*-CD, a cyclic oligosaccharide, can form inclusion complexes with hydrophobic compounds like ECEO, thereby improving their solubility, stability, and controlled release profiles.

A study by Adjali, A. et al. (2022) investigated the encapsulation of ECEO in hydroxypropyl–*β*-cyclodextrin (HP–*β*-CD) and its incorporation into chitosan films. The inclusion complex demonstrated enhanced antioxidant activity compared to free ECEO. Moreover, the chitosan films containing the ECEO–HP–*β*-CD complex exhibited sustained release of ECEO, indicating potential applications in food packaging and preservation [25]. Similarly, research by Sun, X. et al. (2014) focused on the antimicrobial and mechanical properties of *β*-CD inclusion complexes with essential oils. The incorporation of these complexes into chitosan films significantly increased their antimicrobial activities against *Escherichia coli* and *Salmonella* spp., suggesting their suitability for active packaging applications [52].

The literature suggests that other compounds from the ECEO may also contribute to this activity. Studies have demonstrated that eugenyl acetate is active against several bacterial species, including *Listeria monocytogenes*, *Staphylococcus aureus*, *Bacillus subtilis*, *Enterococcus faecalis*, *Bacillus cereus*, *Pseudomonas aeruginosa*, *Serratia marcescens*, *Citrobacter freundii*, *Escherichia coli,* and *Klebsiella pneumonia*. Additionally, humulene has shown efficacy against species of *Pseudomonas*, *Clostridium,* and *Enterococcus* [53,54,55].

## 5. Conclusions

The encapsulation of ECEO in *β*-cyclodextrin (*β*-CD) inclusion complexes has demonstrated significant potential for stabilising and controlling the release of its volatile bioactive components, primarily eugenol. GC-MS and HS-GC-MS analyses confirmed that eugenol is the major constituent of ECEO, and its encapsulation was most efficiently achieved using the kneading method. Other compounds, such as eugenyl acetate, eucalyptol, and *p*-cymene, were also encapsulated—likely due to their favourable polarity and size compatibility with the *β*-CD cavity.

Encapsulation efficiency varied depending on the preparation methods, with kneading being the most efficient, followed by lyophilisation and co-precipitation. The co-precipitation–lyophilisation combined method was the least effective, particularly in the case of eugenol. These results highlight the importance of selecting an appropriate preparation technique to optimise the encapsulation of different bioactive compounds. DSC analysis provided valuable insights into the thermal behaviour of the inclusion complexes, confirming the successful incorporation of eugenol and other components within the cyclodextrin cavity. The observed shifts in thermal transitions indicate that encapsulated compounds were stabilised and protected from volatilisation—an essential feature for practical applications.

Overall, the study underscores the potential of *β*-CD inclusion complexes as a promising strategy to enhance the stability and antimicrobial activity of volatile oils, such as ECEO. These complexes have the potential to modulate the release of natural products, thereby improving their efficacy and stability in pharmaceutical, food, and cosmetic applications. Future studies could further investigate the antimicrobial properties of these complexes, particularly their ability to inhibit the formation of biofilms by multi-resistant strains.

## Figures and Tables

**Table 1:** The chemical composition of ECEO and its *β*-cyclodextrin complexes obtained by various methods resulting from GC-MS analysis.

Compounds	RT (min.) ^1^	RI ^2^	UV ^3^	K ^4^	L ^5^	C-L ^6^	C ^7^
			Relative Area %				
Eugenol	12.84	1358	90.67 ± 1.15	94.71 ± 1.37	93.81 ± 1.33	96.30 ± 1.69	90.99 ± 1.49
Vanillin	13.29	1391	n.d.	0.20 ± 0.01	0.30 ± 0.02	0.26 ± 0.01	1.90 ± 0.04
(E)-*β*-Caryophyllene	13.63	1417	3.98 ± 0.09	0.33 ± 0.03	0.63 ± 0.03	0.50 ± 0.07	2.01 ± 0.04
Humulene	14.03	1449	0.41 ± 0.03	0.06 ± 0.00	0.10 ± 0.00	0.08 ± 0.00	0.27 ± 0.01
Eugenyl acetate	14.84	1513	4.77 ± 0.15	4.66 ± 0.20	5.16 ± 0.13	2.81 ± 0.04	4.68 ± 0.05
Caryophyllene oxide	15.66	1582	0.17 ± 0.02	0.04 ± 0.00	n.d.	0.05 ± 0.00	0.14 ± 0.00
Identified compounds	-	-	100	100	100	100	100

^1^ retention time; ^2^ Kovats indices; ^3^ *Eugenia caryophyllata* essential oil (ECEO); ^4^ K: complexes obtained by kneading; ^5^ L: complexes obtained by lyophilisation; ^6^ C-L: complexes obtained by co-precipitation followed by lyophilisation; ^7^ C: complexes obtained by co-precipitation; n.d.= not detected.

**Table 2:** The chemical composition of ECEO and its *β*-cyclodextrin complexes obtained by various methods resulting from HS-GC-MS analysis.

Compounds	RT (min.) ^1^	RI ^2^	Relative Area (%)				
			ECEO ^3^	K ^4^	L ^5^	C-L ^6^	C ^7^
p-Cymene	7.49	1012	0.10 ± 0.00	0.70 ± 0.03	0.84 ± 0.04	1.57 ± 0.04	1.32 ± 0.07
Limonene	7.56	1016	0.30 ± 0.01	0.73 ± 0.03	0.75 ± 0.02	2.55 ± 0.12	1.29 ± 0.06
Eucalyptol	7.64	1020	0.36 ± 0.03	5.10 ± 0.17	2.45 ± 0.20	6.63 ± 0.20	4.00 ± 0.38
Eugenol	12.72	1349	86.46 ± 0.89	91.44 ± 1.91	92.63 ± 1.84	85.57 ± 1.40	88.75 ± 1.75
Vanillin	13.27	1390	n.d.	n.d.	n.d.	n.d.	0.32 ± 0.03
(E)-*β*-Caryophyllene	13.58	1415	10.96 ± 0.30	0.37 ± 0.02	0.26 ± 0.00	0.17 ± 0.00	0.30 ± 0.03
Humulene	14.01	1455	0.85 ± 0.03	n.d.	n.d.	n.d.	n.d.
Caryophyllene oxide±	14.79	1509	0.98 ± 0.04	1.67 ± 0.05	3.07 ± 0.20	3.52 ± 0.20	4.03 ± 0.29
Identified compounds	7.49	1012	0.10 ± 0.00	0.70 ± 0.03	0.84 ± 0.04	1.57 ± 0.04	1.32 ± 0.07

^1^ retention time; ^2^ Kovats indices; ^3^ *Eugenia caryophyllata* essential oil (ECEO); ^4^ K: complexes obtained by kneading; ^5^ L: complexes obtained by lyophilisation; ^6^ C-L: complexes obtained by co-precipitation followed by lyophilisation; ^7^ C: complexes obtained by co-precipitation; n.d.= not detected.

**Table 3:** The chemical structures of the most abundant constituents identified by GC-MS and HS-GC-MS.

Compound Name			
Eugenol	Eucalyptol	(E)-*β*-Caryophyllene	Eugenyl Acetate
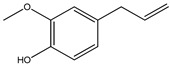	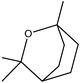	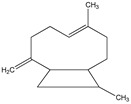	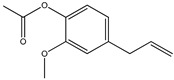

**Table 4:** The entrapment efficiency (EE) of eugenol, eucalyptol, and ECEO in *β*-cyclodextrin by different preparation methods.

Complexation Method	EE (%)		
	Eucalyptol	Eugenol	ECEO
K	90.06 ± 3.3	99.38 ± 5.2	99.40 ± 1.9
C	48.68 ± 2.8	55.01 ± 4.7	69.26 ± 2.4
L	n.d.	77.66 ± 4.9	79.02 ± 2.0
C-L	95.62 ± 3.7	53.96 ± 4.4	60.48 ± 1.9

K: complexes obtained by kneading; C: complexes obtained by co-precipitation; L: complexes obtained by lyophilisation; C-L: complexes obtained by co-precipitation followed by lyophilisation; ECEO: *Eugenia caryophyllata* essential oil; n.d.: not detected.

## Data Availability

The original contributions presented in the study are included in the article; further inquiries can be directed to the corresponding author.

## Supplementary Materials

The following supporting information can be downloaded at: https://www.mdpi.com/article/10.3390/pharmaceutics17070852/s1, Figure S1 (FTIR spectrum of the *β*-Cyclodextrin (A), ECEO–*β*-Cyclodextrin complex (B) and ECEO (C). Legend: *Eugenya cariophyllata* essential oil: ECEO; *β*-Cyclodextrin: *β*-CD; *β*-Cyclodextrin complex with *Eugenya cariophyllata* essential oil: ECEO–*β*-CD); Figure S2 (FTIR spectrum of the *β*-Cyclodextrin (A), EG–*β*-Cyclodextrin complex (B) and EG (C). Legend: Eugenol: EG; *β*-Cyclodextrin: *β*-CD; *β*-Cyclodextrin complex with Eugenol: EG–*β*-CD) and Figure S3 (FTIR spectrum of the *β*-Cyclodextrin (A), E–*β*-Cyclodextrin complex and E (C). Legend: Eucalyptol: E; *β*-Cyclodextrin: *β*-CD; *β*-Cyclodextrin complex with Eucalyptol: E–*β*-CD), as well as Table S1 (Legend: EG: eugenol; E: eucalyptol; ECEO: *Eugenia caryophyllata* essential oil; EG-CD: *β*-Cyclodextrin complex with eugenol; E-CD: *β*-Cyclodextrin complex with eucalyptol; ECEO-CD: *β*-Cyclodextrin complex with *Eugenia caryophyllata* essential oil).

## Abbreviations

The following abbreviations are used in this manuscript:

*β*-CD	*β*-cyclodextrin
EG–*β*-CD	*β*-Cyclodextrin complex with Eugenol
E-CD	*β*-Cyclodextrin complex with eucalyptol
ECEO-CD	*β*-Cyclodextrin complex with *Eugenia caryophyllata* essential oil
C	Complexes obtained by co-precipitation
C-L	Complexes obtained by co-precipitation followed by lyophilisation
DSC	Differential Scanning Calorimetry
E	Eucalyptol
EE	Entrapment Efficiency (EE)
ECEO	*Eugenia caryophyllata* essential oil (clove essential oil)
EG	Eugenol
FTIR	Fourier Transform Infrared Spectroscopy
GC-MS	Gas Chromatography–Mass Spectrometry
GIZD	Growth Inhibition Zone Diameter
HS-GC-MS	Headspace-GC-MS
K	Complexes obtained by kneading
L	Complexes obtained by lyophilisation
MIC	Minimum inhibitory concentration
MBEC	Minimum Biofilm Eradication Concentration
SEM	Scanning Electron Microscopy
