# Supplementary material for: Comparative Evaluation of β-Cyclodextrin Inclusion Complexes with Eugenol, Eucalyptol, and Clove Essential Oil: Characterisation and Antimicrobial Activity Assessment for Pharmaceutical Applications
Source: Pharmaceutics. 2025 Jun 29;17(7):852. doi: 10.3390/pharmaceutics17070852 (PMC12300126; doi:10.3390/pharmaceutics17070852)
Supplement: Supplementary file 1 [file pharmaceutics-17-00852-s001.zip › pharmaceutics-3728455-supplementary.pdf]

Supplementary Material for the Paper entitled "Comparative Evaluation of β-Cyclodextrin Inclusion Complexes with Eugenol, Eucalyptol, and Clove Essential Oil: Characterisation and Antimicrobial Activity Assessment for Pharmaceutical Applications"

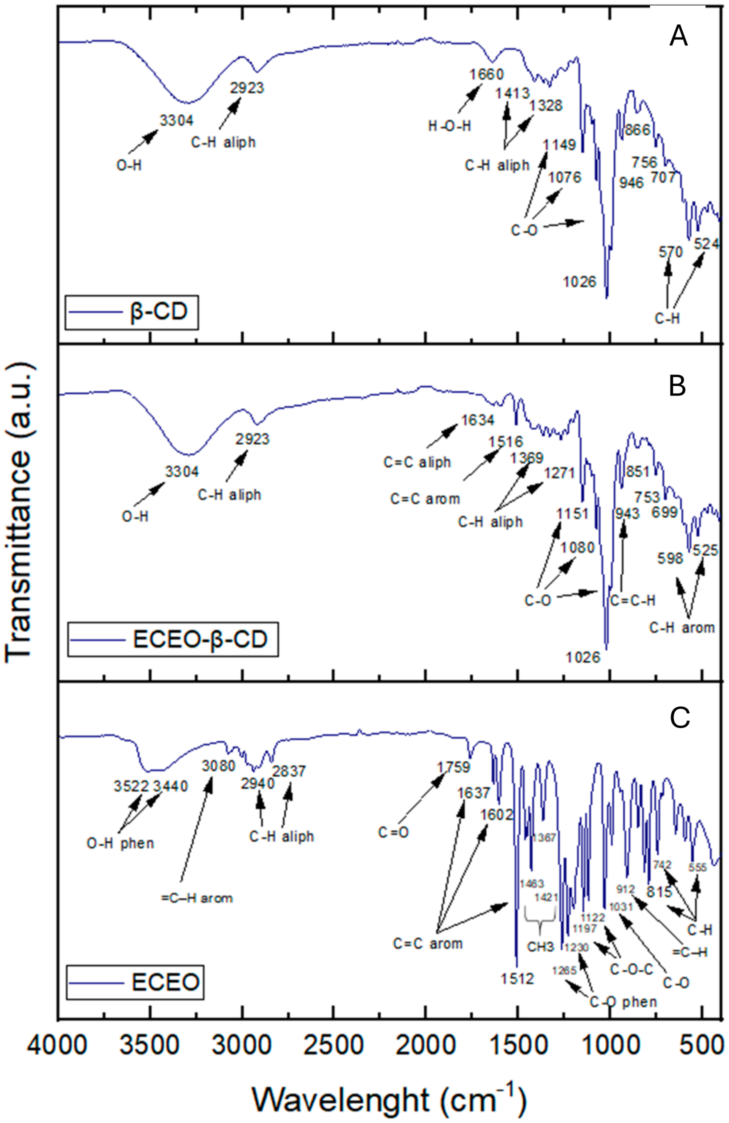

Figure S1. FTIR spectrum of the *β*-Cyclodextrin (A), ECEO- *β*-Cyclodextrin complex (B) and ECEO (C). Legend: *Eugenya cariophyllata* essential oil= ECEO; ***β***-Cyclodextrin= ***β***-CD; ***β***-Cyclodextrin complex with *Eugenya cariophyllata* essential oil = ECEO- ***β***-CD;.

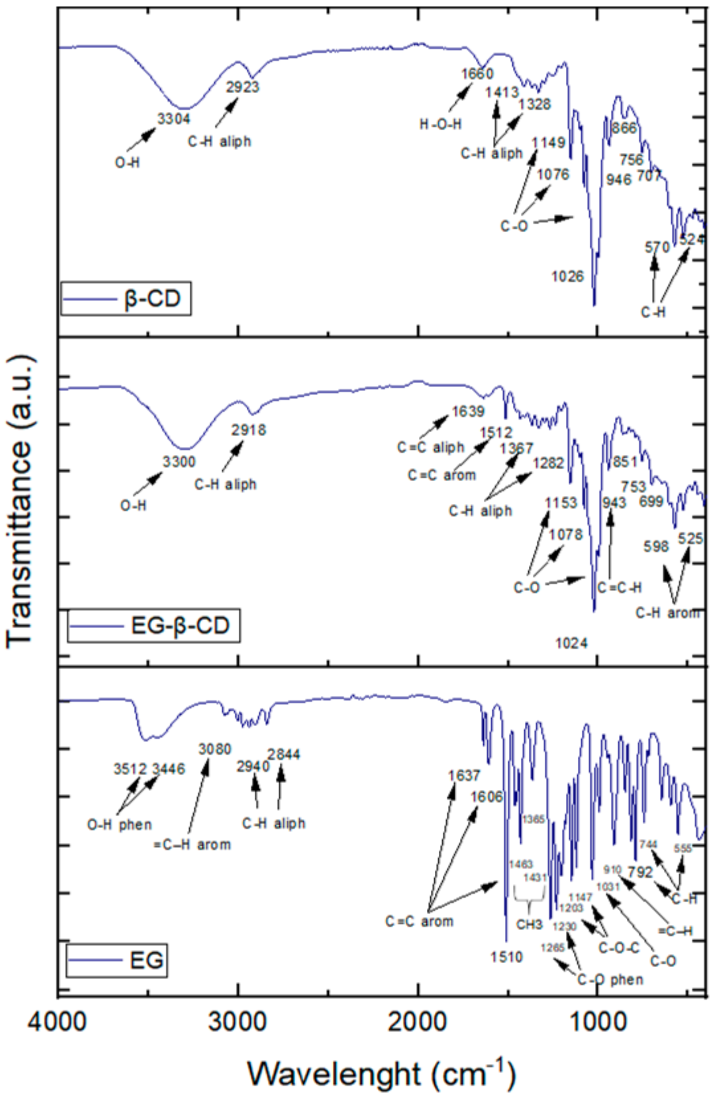

Figure S2. FTIR spectrum of the ***β***-Cyclodextrin (A), EG- ***β***-Cyclodextrin complex (B) and EG (C). Legend: Eugenol = EG; ***β***-Cyclodextrin= ***β***-CD; ***β***-Cyclodextrin complex with Eugenol= EG- ***β*** -CD;

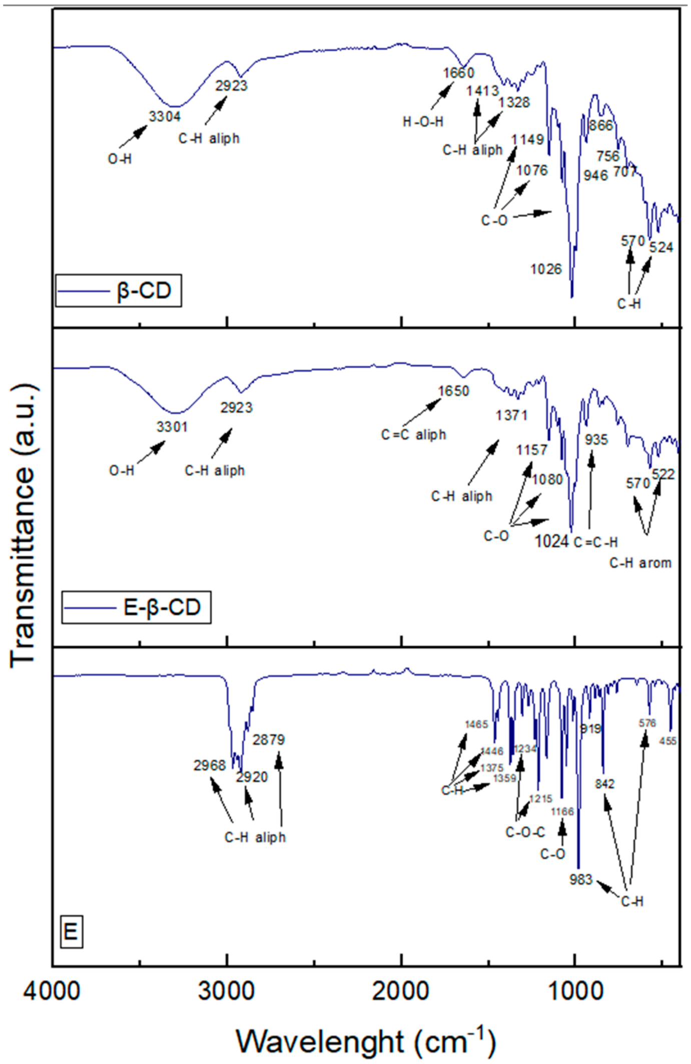

Figure S3. FTIR spectrum of the ***β***-Cyclodextrin (A), E- ***β***-Cyclodextrin complex and E (C). Legend: Eucalyptol = E; ***β***-Cyclodextrin= ***β***-CD; ***β***-Cyclodextrin complex with Eucalyptol = E- ***β*** -CD;

Table S1. The influence of cyclodextrin complexes with antimicrobial compounds on selected pathogenic strains.

Strains	Samples							
	EG	E	ECEO	EG-CD	E-CD	ECEO-CD	Gentamicin	DMSO
**Gram-positive bacteria**								
***E. faecalis* ATCC 29212**	++	-	++	+	-	++	++	-
***S. aureus* ATCC 25923**	++	-	++	++	-	++	+++	-
B. subtilis ATCC 21332	++	-	++	++	-	++	+++	-
**Gram-negative bacteria**								
***E. coli* ATCC 25922**	+	++	+	+	+	++	+++	-
E. cloacae	++	+	++	++	+	++	+++	-
***P. aeruginosa* ATCC 27853**	++	++	++	+	++	++	+++	-

Legend: EG = eugenol; E = eucalyptol; ECEO = *Eugenia caryophyllata* essential oil; EG-CD = *β*-Cyclodextrin complex with eugenol; E-CD = *β*-Cyclodextrin complex with eucalyptol; ECEO-CD = *β*-Cyclodextrin complex with *Eugenia caryophyllata* essential oil.

(-) GIZD=0; (+) GIZD between 5 and 10 mm; (++) GIZD between 10 and 20 mm; (+++) GIZD between 20 and 30 mm

E. faecalis - Enterococcus faecalis; S. aureus - Staphylococcus aureus; B. subtilis - Bacillus subtilis; E. coli - Escherichia coli; E. cloacae - Enterobacter cloacae; P. aeruginosa - Pseudomonas aeruginosa
